# Ear Disease

**Published:** 1890-09-20

**Authors:** 


### SURGERY.

### EAR DISEASE.

Mr. Arthur Barker's Hunterian lectures on "Intra-cranial inflammations, starting in the temporal bone" delivered some time ago at the College of Surgeons, have recently been published in a separate form. The author refers chiefly to inflammatory changes within the cranium and their complications due to disease of the temporal bone. That suppurative disease of this bone is common, every medical man is aware. But Mr. Barker thinks that a correct notion as to its dangers is not entertained by the profession, nor by the general public. One of the causes why the affection is so rife appears to Mr. Barker to be the neglect of medical men of the study of the pathology and clinical features of the commonest ear disease, which is relegated to the aurist after delay has quadrupled the difficulties. The question of the patient's welfare is determined by the treatment during the first hours of the attack. When we look for the points at which primary inflammation arises in the temporal bone, the internal ear and the external meatus may practically be left out of consideration. From 11,654 cases it appears that 66·9 per cent. are affections of the middle ear, a large proportion of which are suppurative affections. Mr. Barkerthinks it probable, that in the production of that kind of *otitis media* which is accompanied by cattarh of the naso-pharynx, bacilli play an important part. There is a great difference between fœtid and non-fœtid discharge. In the former cocci and bacilli are found. In the latter cocci alone. It has been commonly taught that a bad smell from the ear is important as regards prognosis. Mr. Barker questions this. The explanation is found in pathogenic cocci being found in the non-fœtid discharges, and a preponderance of merely saprophytic bacilli in the fœtid. We must not therefore think less seriously of a discharge because it is odourless. If intra-cranial inflammations due to ear disease depend upon the presence of pathogenic organisms, it is important to ascertain in what way they gain access to the interior of the skull. That they travel direct seems proved by the fact that auro-intra-cranial disease is almost always on the same side as the affected ear. Primary acute inflammation is not followed by intra-cranial complications nearly so frequently as chronic ear disease. The walls of the tympanum become more or less carious, but there are arteries and veins which communicate with those of the dura mater, and may become the channels of infection. It has been noticed that patients who have had ear discharge for a long time suddenly become ill after measures have been adopted for clearing out the middle ear. Cases of the kind have led to the idea that manipulations on a septic cavity close to some of the largest vessels of the body, set free dangerous material, which may be carried to the lungs or elsewhere, producing pneumonia or other ailments. Mr. Barker thinks that bacteriology helps us to understand these anomalous cases, in which ear disease gives rise to deep seated abscess in the cerebellum or brain, while the surfaces of the convolutions appear healthy, organisms being conveyed by minute radicles running to the part. As far as the dura mater is concerned, it is not difficult to understand, inasmuch as there is free inosculation between the arterioles of the ear and the dura. A case of thrombosis of the internal carotid artery as the result of intra-tympanic inflammation, has been recorded by Dr. Gairdner. Attention has been called to cases of tympanic suppuration complicated with optic neuritis. It is possible that damage to the carotid plexus of the sympathetic from intra-aural inflammation may produce vasomotor disturbances in the optic nerve and fundus. The most prominent lesions observed in connection with suppurative aural disease are cerebral abscess, meningitis, cerebellar abscess, pyæmia, phlebitis of the lateral sinus, and occasionally marasmus. Meningitis and pyæmia are most to be feared. Symptoms, of course, vary considerably but are all detailed by Mr. Barker. As regards treatment, the author insists on stringent prophylaxis. The disease should be attacked vigorously at the onset. Under well-directed antiseptic treatment, suppurative otitis media may be arrested. But in chronic cases operative procedure is necessary. As long as septic matter is pent up in the temporal bone or inside the skull, other means are waste of time. The only treatment which affords hope of success is to gain access to the focus of injection in the middle ear, and to thoroughly cleanse it. Next, to search for the secondary inflamed area within the skull and cleanse and drain it likewise. "Antiseptic irrigation and fomentation are of the utmost value, once the foci of inflammation have been reached by removal of bone, but before this has been done must obviously be quite ineffectual whether the pus lie in the mastoid cells, the sulcus lateralis, or the brain." In a case of phlebitis of the lateral sinus it is necessary to make a free opening through the mastoid bone into the middle ear. Cleansing hyodoform emulsion and boracic fomentations are then used. If the symptoms are not relieved the mastoid process should be gouged away until the membranous lateral sinus is well exposed. Recovery has followed these operations. Methods of performing operations for other complications are also detailed, which we have not space to notice. Suffice it to say that the operative achievements of recent years, have amply demonstrated that even with advanced inter-cranial disease of the kind recovery is not impossible, if only a free escape of septic matter is secured. Dr. Macewen, of Glasgow, recently opened the cerebellum, giving exit to four ounces of pus, and the patient recovered. Mr. Barker's book should be studied with attention.

A paper by Dr. Adolf Bronner, surgeon to the Bradford Eye and Ear Infirmary (*British Medical Journal*), treats "On massage of the membrana tympani and the ossicula, in the treatment of chronic catarrh of the middle ear." Dr. Bronner says the most common cause of deafness is as some call it *chronic non-suppurative catarrh*, or *chronic dry catarrh*, or the old-fashioned name, "obstruction of the eustachian tube." Although Dr. Bronner does not say so, obstruction of the eustachian tube is commonly owing to the extension of the catarrh from the pharynx. From the obstacle to the admission of air the balance of pressure on the inner and outer sides of the tympanic membrane is disturbed. Professor Delstanche, of Brussels, has suggested an instrument by which the air in the external meatus is alternately compressed and evacuated, thus moving the drumhead and ossicula up and down. Professor Lucae recommends what he terms a pressure probe, which consists of a probe with an enlarged concave end attached to a weak spiral spring. Dr. Bronner states he has used Delstanche's and Lucae's instruments in many cases. At first he used Delstanche's method alone, but without good results. Latterly he always uses first the eustachian catheter, then Delstanche's instrument, and then Politzer's bag, at one sitting. The results have been very good. The author advises early treatment, and protests against the idea that this variety of ear affection is incurable. Dr. Bronner does not say anything about constitutional treatment; but we think the condition generally requires constitutional treatment, and also local treatment of the pharynx. It may be associated with alcoholic excess, with the strumous diathesis, or with rheumatic, gouty, or syphilitic conditions, which should be treated if present In connection with the above, we notice Dr. John Ward Cousins', of Portsmouth, "new artificial membrana tympani." The artificial drumhead is reported to be especially useful in cases of perforation, also in other alterations of the membrane involving abnormalities of tension, ossicular changes and atrophy of the drum, and in inflammatory thickening of the membrane lining the tympanic cavity. A large number of different kinds of artificial drumheads have been proposed since Itard, an Italian aurist, some five and thirty years ago introduced the practice of inserting cotton wool. But the essential qualities of a good artificial drumhead are that it must decidedly improve the hearing power; it must be easily placed and removed; it must fit the varying capacity of the meatus; it should maintain the moisture of the exposed tympanic cavity; it should be a convenient vehicle for the application of remedies; the cost should be trifling. Dr. Cousins' antiseptic artificial drum is shaped like a hat, is firm enough to retain its shape, yet flexible enough to cause no sensation by its presence, and is made of various sizes. When in position the crown rests near the tympanic membrane, the brim upon the meatal wall. A short ribbon or handle attached to the edge rests behind the tragus. It is a matter of importance that the artificial membrane should be selected to suit the shape and capacity of the external ear. The artificial drumhead may be regarded as an aid to hearing, also as an ear protector, and when impregnated with various substances as a local antiseptic remedy. As a general rule, the help afforded by the artificial drumhead is more pronounced in perforation than in any other form of middle-ear disease. Often good results may be obtained by simply adjusting the drumhead and replacing it as often as necessary. In many instances other measures must be practised. The external meatus must be kept clean by washing, and the ear must be thoroughly deodorised before the introduction of the artificial membrane. Parents should be taught how to do this. In cases of profuse otorrhœa, Dr. Cousins does not recommend the use of the artificial membrane, until the discharge has been relieved and excoriations around the meatus have been successfully treated. Care must be taken that the purulent secretion from the diseased tympanum is not mechanically pent up. Plugging the ear with cotton wool, or any other substance, is a dangerous practice.

